# CMOS Fixed Pattern Noise Elimination Based on Sparse Unidirectional Hybrid Total Variation

**DOI:** 10.3390/s20195567

**Published:** 2020-09-28

**Authors:** Tao Zhang, Xinyang Li, Jianfeng Li, Zhi Xu

**Affiliations:** 1 Key Laboratory of Adaptive Optics, Institute of Optics and Electronics, Chinese Academy of Sciences, Chengdu 610209, China; ztao@ynao.ac.cn; 2 School of Optoelectronic Information, University of Electronic Science and Technology, Chengdu 611731, China; lijianfeng@uestc.edu.cn; 3 Astronomical Technology Laboratory, Yunnan Observatory, Chinese Academy of Sciences, Kunming 650216, China; xuzhi@ynao.ac.cn

**Keywords:** FPN, sparse, total variation, anisotropy, characteristic

## Abstract

With the improvement of semiconductor technology, the performance of CMOS Image Sensor has been greatly improved, reaching the same level as that of CCD in dark current, linearity and readout noise. However, due to the production process, CMOS has higher fix pattern noise than CCD at present. Therefore, the removal of CMOS fixed pattern noise has become the research content of many scholars. For current fixed pattern noise (FPN) removal methods, the most effective one is based on optimization. Therefore, the optimization method has become the focus of many scholars. However, most optimization models only consider the image itself, and rarely consider the structural characteristics of FPN. The proposed sparse unidirectional hybrid total variation (SUTV) algorithm takes into account both the sparse structure of column fix pattern noise (CFPN) and the random properties of pixel fix pattern noise (PFPN), and uses adaptive adjustment strategies for some parameters. From the experimental values of PSNR and SSM as well as the rate of change, the SUTV model meets the design expectations with effective noise reduction and robustness.

## 1. Introduction

**Figure 1:**
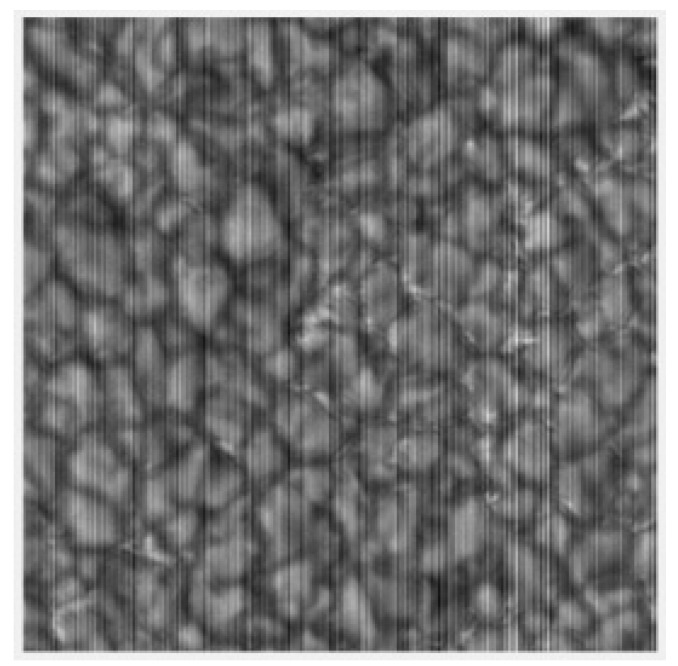
Image of the Sun’s quiet zone.

For a long time, CCD has had advantages of high quantum efficiency, high sensitivity, low dark current, good consistency and low noise compared with CMOS image sensors (CISs). However, in recent years, with the development of large-scale integrated circuit technology, the photoelectric characteristics of CIS have been greatly improved. Most of the disadvantages of CIS have been improved to almost the same level as those of CCD. At the same time, CIS has quickly taken market share from CCD with its advantages of low cost and low power consumption and CIS has rapidly entered various fields, such as consumer electronics, space, medicine, astronomy, remote sensing, military and so on. In astronomy, major Chinese observatories have begun the wide application of CIS, such as the high-resolution imaging system of the 1-m infrared solar tower of the Chinese Academy of Sciences Yunnan Astronomical Observatory, which uses CIS as its imaging terminal. In the final imaging results, it is found that there are distinct column stripes in the images, as shown in Figure 1. These column stripes are caused by a fixed pattern noise (FPN) characteristic of CIS. The generation of FPN is mainly due to the mismatch of CIS pixel structure and readout structure [1,2]. Generally speaking, FPN consists of two parts: pixel fixed pattern noise (PFPN) and column fixed pattern noise (CPFN). According to the relevant research, the influence of structural rule CFPN on image quality is dozens of times of random noise [3]. Therefore, in order to eliminate the impact of CFPN on image quality, many scholars have studied this problem.

Generally speaking, there are three types of methods to eliminate CFPN: calibration-based, statistics-based, and scene-based. Calibration-based methods have the characteristics of simple calculation and high correction accuracy, and are widely used in engineering applications. The most commonly used method is the two-point calibration method [4,5]. The corresponding photoelectric curves of CIS show a certain degree of non-linearity. Simple FPN correction using two-point equation may have a problem of low correction accuracy. In order to improve the accuracy of correction caused by non-linear problems, Hong and Zhou et al. [6,7] proposed a piecewise correction method. Although the piecewise correction can solve the problem of low correction accuracy well, it brings the problem of computational complexity. In order to balance the compromise between accuracy and calculation, Rui et al. [8] proposed the S-curve method. This method can improve the correction accuracy very well and rarely increases the computational complexity. However, the biggest problem of calibration methods is that with the lapse of working time and working environment changes, there will be a mismatch of calibration parameters, which requires repeated periodic recalibration, and since the calibration process involves special experimental environments and equipment, suitable conditions cannot be provided in most working scenarios.

The statistical-based methods are methosd to correct non-uniformity based on the statistical characteristics of each sensor. The typical statistical methods include the histogram matching method [9], moment matching method [10], and constant statistical method [11]. The histogram matching method is more suitable for multi-sensor imaging systems where the incident radiation distribution of each sensor is the same. In principle, a histogram matching method eliminates FPN by adjusting the histogram distribution of each sensor to a reference distribution. For single CMOS imaging, each column can be approximately regarded as an independent sensor. The FPN is eliminated by adjusting the histogram of each column to the global histogram. However it is difficult to ensure that the radiation distribution of each column of sensors is consistent due to the illumination angle. If this condition cannot be met, there will be a large error in the correction results. Moment matching methods mainly involve the statistics of the mean value and variance of the corresponding pixels of multiple frames, and then the mean and variance of each pixel point are transferred to the reference value to complete the FPN elimination. This method is more suitable for matching images with a single background, but for images with complex details, the matching error will be larger. In general, this type of method has to satisfy many prior assumptions in order to achieve good results, and these assumptions are hard to satisfy in non-laboratory scenarios.

The scene-based methods start from the image itself and use the prior information of the image to complete the denoising of CFPN. Typical methods are the low-pass filter [12,13], wavelet filter [14,15,16,17,18,19,20] and total variation methods [21,22,23,24,25,26,27,28,29,30,31,32,33,34]. The low-pass filtering method is more suitable for CFPN with periodicity. In the non-periodic case, because the energy of the stripes is distributed throughout the frequency range, it is very easy to lose the details of the image when using low-pass filtering. Generally speaking, the denoising effect of the wavelet method is better than that of the simple low-pass filter, but it is difficult to select the threshold value in the filtering process. It is difficult to completely separate the CPFN from the clear image. Since the total variation method was proposed in 1992, it has become a research hotspot because of its excellent noise removal effect and edge preservation ability. In recent years, many scholars have also successfully applied the variational method to the removal of FPN, and achieved good results. Typical methods are described in [21,22,23,24,25]. In summary, most of the above methods remove CFPN only from the smoothness of the image, but seldom consider the unique structure of CFPN, therefore, these methods have room for further improvement. In recent years, some scholars have begun to separate FPN from clear images by incorporating the low rank or sparse properties of CFPN stripes into the traditional total variation method. Typical examples of such methods are described in [26,27,28,29]. However, PFPN is seldom considered by these methods, so there is still obvious random noise after de-noising. Our method considers not only the structural characteristics of CPFN, but also PFPN. Therefore, both CFPN and PFPN can be removed at the same time. Overall, our method is a more comprehensive method of noise reduction. The process of image denoising by total variation method is essentially a process of solving convex optimization problems. Whether this method can effectively remove noise mainly involves three issues. The first is the establishment of a reasonable optimization model, the second is the solution of the model, and the last is the selection of reasonable regular coefficients. This article is also from these three aspects to elaborate our methods.

## 2. Problem Analysis, Model Establishment and Solution

**Figure 2:**
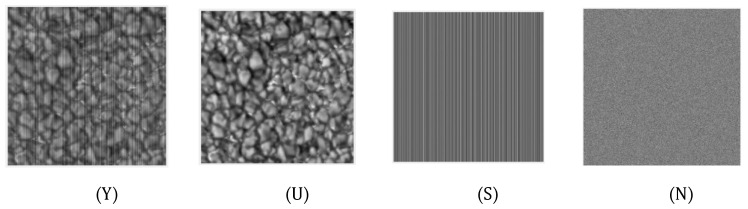
Noise Composition.

First, assume that the FPN is distributed vertically, as shown in Figure 2. The noise image Y can be decomposed into a clear image U, column fix pattern noise S and pixel fix pattern noise N. The mathematical representation is shown in Equation (1) and the decomposition effect is shown Figure 2.

(1) Y=U+S+N

By analyzing Figure 2, the following regular properties can be obtained:

- A regular term for ${\left\|\frac{\partial S}{\partial y}\right\|}_{1}$ that can be generated by the directional nature of S.
- Regular terms of $\|\frac{\partial Y}{\partial y}-{\frac{\partial U}{\partial y}\|}_{1}$ can be generated from the vertical gradient similarity of Y and U.
- Regular terms of ${\left\|\frac{\partial U}{\partial x}\right\|}_{1}$ can be generated from the horizontal continuity of the clear image U.
- Regular terms of ${\left\|Y-U-S\right\|}_{2}^{2}$ can be generated from the structural similarity before and after denoising.

Through the above analysis of the problem, it is found that the whole CFPN stripe removal problem can be described by the optimization equation shown in (2)

(2) $$\min E\left(U,S\right)=\frac{1}{2}\|Y-U-{S\;\|}_{2}^{2}+a_{2}{\left\|\frac{\;\partial U}{\partial x}\right\|}_{1}\;s.t.{\left\|\frac{\partial S}{\partial y}\right\|}_{1}=0\;\|\frac{\partial Y}{\partial y}-{\frac{\partial U}{\partial y}\|}_{1}=0$$

Equation (2) is an optimization equation with multiple constraints, where Y is the input noise image, U is the expected clear image, S is CFPN, N is PFPN. According to the optimization theory, KKT multiplier method can be used to transform the constrained equation into unconstrained equation, as shown in formula (3).

(3) $$\min\;E\left(U,S\right)=\frac{1}{2}{\left\|Y-U-S\right\|}_{2}^{2}+a_{2}{\left\|\frac{\;\partial U}{\partial x}\right\|}_{1}+a_{3}{\left\|\frac{\partial S}{\partial y}\right\|}_{1}+a_{4}\|\frac{\partial Y}{\partial y}-{\frac{\partial U}{\partial y}\|}_{1}$$

where $a_{2},a_{3},a_{4}$ is the regular coefficient. Equation (3) belongs to the multivariable optimization problem. At present, Bregman and ADMM are two commonly used methods to solve multivariable optimization. ADMM algorithm is used in this paper. By this method, Equation (3) can be decomposed into sub problems and solved one by one, which greatly simplifies the difficulty of solving the optimal solution. In ADMM algorithm, variable splitting is used to solve 1 norm. In the process of variable splitting, several auxiliary variables H, J, K, L are introduced. Let $H=\frac{\;\partial U}{\partial x}$, $J=\frac{\partial S}{\partial y}$, $K=\frac{\partial Y}{\partial y}-\frac{\partial U}{\partial y}$. Thus, Equation (3) can be equivalent to formula (4)

(4) $$\min\;E\left(U,S\right)=\frac{1}{2}{\left\|Y-U-S\right\|}_{2}^{2}+a_{2}{\left\|H\right\|}_{1}+a_{3}{\left\|J\right\|}_{1}+a_{4}{\left\|K\right\|}_{1}\;s.t.\;H=\frac{\;\partial U}{\partial x}\;,\;J=\frac{\partial S}{\partial y}\;,\;K=\frac{\partial Y}{\partial y}-\frac{\partial U}{\partial y}$$

According to the augmented Lagrange equation, the constrained Equation (4) can be transformed into the unconstrained Equation (5).

(5) $$E\left(U,S,H,J,K\right)=\frac{1}{2}{\left\|Y-U-S\right\|}_{2}^{2}+a_{2}{\left\|H\right\|}_{1}+a_{3}{\left\|J\right\|}_{1}+a_{4}{\left\|K\right\|}_{1}\;+\langle R_{2},H-\;\frac{\;\partial U}{\partial x}\rangle +\langle R_{3},J-\;\frac{\partial S}{\partial y}\rangle +\langle R_{4},K-\left(\frac{\partial Y}{\partial y}-\frac{\partial U}{\partial y}\right)\rangle \;+\frac{w_{2}}{2}{\left\|H-\;\frac{\;\partial U}{\partial x}\right\|}_{2}^{2}+\frac{w_{3}}{2}{\left\|J-\;\frac{\partial S}{\partial y}\right\|}_{2}^{2}+\frac{w_{4}}{2}{\left\|K-\left(\frac{\partial Y}{\partial y}-\frac{\partial U}{\partial y}\right)\right\|}_{2}^{2}$$

where $R_{2},R_{3},R_{4}$ is a regular matrix, $a_{2},a_{3,\;}a_{4}$ is the regular coefficient. Equation (6) is obtained by combining and simplifying Equation (5).

(6) $$E\left(U,S,H,J,K\right)=\frac{1}{2}{\left\|Y-U-S\right\|}_{2}^{2}+a_{2}{\left\|H\right\|}_{1}+a_{3}{\left\|J\right\|}_{1}+a_{4}{\left\|K\right\|}_{1}\;+\frac{w_{2}}{2}{\left\|H-\;\frac{\;\partial U}{\partial x}+\frac{R_{2}}{w_{2}}\right\|}_{2}^{2}+\frac{w_{3}}{2}{\left\|J-\;\frac{\partial S}{\partial y}+\frac{R_{3}}{w_{3}}\right\|}_{2}^{2}+\frac{w_{4}}{2}{\left\|K-\left(\frac{\partial Y}{\partial y}-\frac{\partial U}{\partial y}\right)+\frac{R_{4}}{w_{4}}\right\|}_{2}^{2}$$

According to ADMM algorithm, the optimization of Equation (6) can be decomposed into several sub variables to optimize one by one. The following is the concrete calculation process as follows.

1. Subproblems about U

(7) $$\begin{array}{c} \underset{U}{\min}\;E\left(U,S,H,J,K,L\right)=\frac{1}{2}{\left\|Y-U-S\right\|}_{2}^{2}+\frac{w_{2}}{2}{\left\|H-\frac{\;\partial U}{\partial x}+\frac{R_{2}}{w_{2}}\right\|}_{2}^{2}\\ +\frac{w_{4}}{2}{\left\|K-\left(\frac{\partial Y}{\partial y}-\frac{\partial U}{\partial y}\right)+\frac{R_{4}}{w_{4}}\right\|}_{2}^{2} \end{array}$$

Solve the extremum of the equation (7)

$$\frac{\partial E\left(U,S,H,J,K\right)}{\partial U}=0$$

$$U+w_{2}\frac{\partial^{2}U}{\partial x^{2}}+w_{4}\frac{\partial^{2}U}{\partial y^{2}}=a_{1}\left(Y-S\right)+w_{2}\left(\frac{\partial H}{\partial x}+\frac{1}{w_{2}}\frac{\partial R_{2}}{\partial x}\right)-w_{4}\left(\frac{\partial K}{\partial y}-\frac{\partial^{2}Y}{\partial y^{2}}+\frac{1}{w_{4}}\frac{\partial R_{4}}{\partial y}\right)$$

Fourier transform can be used to solve the above equation, and fast Fourier transform (FFT) can be used to speed up the solution process. The following equation can be obtained by Fourier transform on both sides of the upper equation:

$$ℱ\left(U\right)\left(1+w_{2}ℱ\left(\frac{\partial^{2}}{\partial x^{2}}\right)+w_{4}ℱ\left(\frac{\partial^{2}}{\partial y^{2}}\right)\right)=ℱ\left(Y-S\right)+w_{2}ℱ\left(\frac{\partial }{\partial x}\right)ℱ\left(H\right)+ℱ\left(\frac{\partial }{\partial x}\right)ℱ\left(R_{2}\right)$$

$$-\;w_{4}ℱ\left(\frac{\partial }{\partial y}\right)ℱ\left(K\right)+w_{4}ℱ\left(\frac{\partial^{2}}{\partial y^{2}}\right)ℱ\left(Y\right)-ℱ\left(\frac{\partial }{\partial y}\right)ℱ\left(R_{4}\right)$$

where ℱ is a Fourier transform operator:

$$ℱ\left(U\right)=\frac{ℱ\left(Y-S\right)+w_{2}ℱ\left(\frac{\partial }{\partial x}\right)ℱ\left(H\right)+ℱ\left(\frac{\partial }{\partial x}\right)ℱ\left(R_{2}\right)-{\;w}_{4}ℱ\left(\frac{\partial }{\partial y}\right)ℱ\left(K\right)+w_{4}ℱ\left(\frac{\partial^{2}}{\partial y^{2}}\right)ℱ\left(Y\right)-ℱ\left(\frac{\partial }{\partial y}\right)ℱ\left(R_{4}\right)}{1+w_{2}ℱ\left(\frac{\partial^{2}}{\partial x^{2}}\right)+w_{4}ℱ\left(\frac{\partial^{2}}{\partial y^{2}}\right)}$$

(8) $$U={ℱ}^{-1}ℱ\left(U\right)$$

where ${ℱ}^{-1}$ is an inverse Fourier transform operator:

2. Subproblems about S

$$\underset{S}{\min}\;E\left(U,S,H,J,K,L\right)=\frac{1}{2}{\left\|Y-U-S\right\|}_{2}^{2}+\frac{w_{3}}{2}{\left\|J-\;\frac{\partial S}{\partial y}+\frac{R_{3}}{w_{3}}\right\|}_{2}^{2}$$

$$\frac{\partial E\left(U,S,H,J,K,L\right)}{\partial S}=0$$

$$S+{\;w}_{3}\;\frac{\partial^{2}S}{\partial y^{2}}+w_{5}\frac{\partial^{2}S}{\partial y^{2}}=\left(Y-U\right)+w_{3}\left(\frac{\partial J}{\partial y}+\frac{1}{w_{3}}\frac{\partial R_{3}}{\partial y}\right)$$

$$ℱ\left(S\right)=\frac{ℱ\left(Y-U\right)+{\;w}_{3}ℱ\left(\frac{\partial }{\partial y}\right)ℱ\left(J\right)+ℱ\left(\frac{\partial }{\partial y}\right)ℱ\left(R_{3}\right)}{1+w_{3}ℱ\left(\frac{\partial^{2}}{\partial y^{2}}\right)}$$

(9) $$S={ℱ}^{-1}ℱ\left(S\right)$$

3. Subproblems about H

$$\underset{H}{\min}\;E\left(U,S,H,J,K,L\right)==a_{2}{\left\|H\right\|}_{1}+\frac{w_{2}}{2}{\left\|H-\frac{\;\partial U}{\partial x}+\frac{R_{2}}{w_{2}}\right\|}_{2}^{2}$$

A soft threshold method can be used to solve the extreme value of H:

(10) $$H=\mathrm{SoftThreshold}\left(\frac{\;\partial U}{\partial x}-\frac{R_{2}}{w_{2}},\frac{2a_{2}}{w_{2}}\right)$$

where SoftThreshold(u,a)=sign(u)max{|u|−a,0}.

4. Subproblems about J

$$\underset{J}{\min}\;E\left(U,S,H,J,K,L\right)=a_{3}{\left\|J\right\|}_{1}+\frac{w_{3}}{2}{\left\|J-\frac{\partial S}{\partial y}+\frac{R_{3}}{w_{3}}\right\|}_{2}^{2}$$

(11) $$J=\mathrm{SoftThreshold}\left(\frac{\partial S}{\partial y}-\frac{R_{3}}{w_{3}},\frac{2a_{3}}{w_{3}}\right)$$

Similar to step 3, the soft threshold method is used to solve the norm. The definition of soft threshold function is the same as that in step 3.

5. Subproblems about K

$$\underset{K}{\min}\;E\left(U,S,H,J,K,L\right)=a_{4}{\left\|K\right\|}_{1}+\frac{w_{4}}{2}{\left\|K-\left(\frac{\partial Y}{\partial y}-\frac{\partial U}{\partial y}\right)+\frac{R_{4}}{w_{4}}\right\|}_{2}^{2}$$

(12) $$K=\mathrm{SoftThreshold}\left(\left(\frac{\partial Y}{\partial y}-\frac{\partial U}{\partial y}\right)-\frac{R_{4}}{w_{4}},\frac{2a_{4}}{w_{3}}\right)$$

6. Update Lagrange Multiplier $R_{2},R_{3},R_{4}$

Updates to Lagrange multipliers $R_{2},R_{3},R_{4}$ are mainly accomplished by applying a gradient-rise method in dual space:

(13) $$R_{2}=R_{2}+w_{2}‗\left(H-\;\frac{\;\partial U}{\partial x}\right)$$

(14) $$R_{3}=R_{3}+w_{3}‗\left(J-\;\frac{\partial S}{\partial y}\right)$$

(15) $$R_{4}=R_{4}+w_{4}‗\left(K-\left(\frac{\partial Y}{\partial y}-\frac{\partial U}{\partial y}\right)\right)$$

where $w_{2},w_{3},w_{4}$ is the iteration step.

In the Algorithm 1, Y is the input noise image. $R_{2},R_{3},R_{4}$ is a Lagrange multiplier. H, J, K are split variables. $w_{2},w_{3},w_{4}$ is the iteration step size when updating Lagrange multiplier $R_{2},R_{3},R_{4}$. $a_{2},a_{3},a_{4}$ is the Lagrange multiplier of the split variable. Num is the number of iteration steps.
**Algorithm 1** Sparse Total Variationnal Destripe (SUTV)1. Get image Y with FPN2. The initial matrix $U=0,\;S=0,N=0,{\;R}_{2}=0,{\;R}_{3}=0,{\;R}_{4}=0,\;H=0,\;J=0,\;K=0$3. Initial optimization factor $\;\;a_{2},a_{3},a_{4},w_{2},w_{3},w_{4},\;N$4. For n = 1:N do**5.** Calculating the optimal solution of U via Fourier Transformation by (8)**6.** Calculating the optimal solution of S via Fourier Transformation by (9)**7.** calculating H, J, K, through soft thresholds by (10)–(12)**8.** Updata ${\;R}_{2},R_{3},R_{4}\;\mathrm{by}\;\mathrm{method}\;\mathrm{of}\;\mathrm{dual}\;\mathrm{gradient}\;\mathrm{rise}\;\mathrm{by}\;(13)–(15)$9. End for10. Separate clear image U and stripe S


## 3. Experimental Results and Discussion

### 3.1. Experimental Environment

To better show the results of the experiment, we adjusted the gray scale of the original image to [0,255], and zoomed the size of the image to 512 × 512.The standard deviation range of FPN noise is set to [0,20]. This range of choices is based on the PRUN strength of existing consumer CIS and research CIS. For scientific CIS, PRNU is about 0.5%, while for consumer CIS, PRNU is about 2%. Therefore, the [0,20] range we selected fully covers the FPN intensity that we may encounter in general. To fully demonstrate the effectiveness of our proposed algorithm (SUTV), we compare it with the competing methods from both simulated and real data. These competing methods are GSUTV [29], wavelet [30], UTV [31], ${ℒ}_{0}$ [32], SILR [33], ASSTV [34] and the recommended SUTV methods in this paper. For the quality assessment of various methods after noise removal, we use subjective evaluation and objective evaluation. In the subjective evaluation, we used the human eye to observe the result after noise removal and Mean Cross-trace curve comparison. In the objective evaluation, we use PSNR and SSIM as two indicators. The non-adaptive Lagrange multiplier involved in our algorithm is determined by hard tuning. The Lagrange parameters involved in other optimum-based competition methods are also adjusted to their optimum state in a hard-tuned way. The Lagrange multipliers in these methods are adjusted when the noise intensity is σ=12. The wavelet transform method adjusts the filter threshold to the optimal value when the db7 base wave and three levels of wavelet decomposition are involved.

### 3.2. Simulation Experiment

In the simulation experiment, we simulate images with different intensities of CFPN noise. In order to truly restore the zero mean Gaussian distribution of CFPN, we set the mean value of CFPN to be μ=0, the variance σ=[0:20], and the location of the CFPN distribution is random. The results of the experiment can be evaluated both subjectively and objectively. Subjectively, we will make a subjective judgment based on the denoised image and Mean Cross-trace curve of the denoised image. Objectively, we evaluated the imaging quality by two quantitative indicators: PSNR and SSIM. For the selection of experimental images, we mainly select two types of images which are more representative in astronomical observation, one is a relatively single structure of the solar sphere picture, the other is a picture of the sun’s active regions with rich details.

#### 3.2.1. Subjective Evaluation

**Figure 3:**
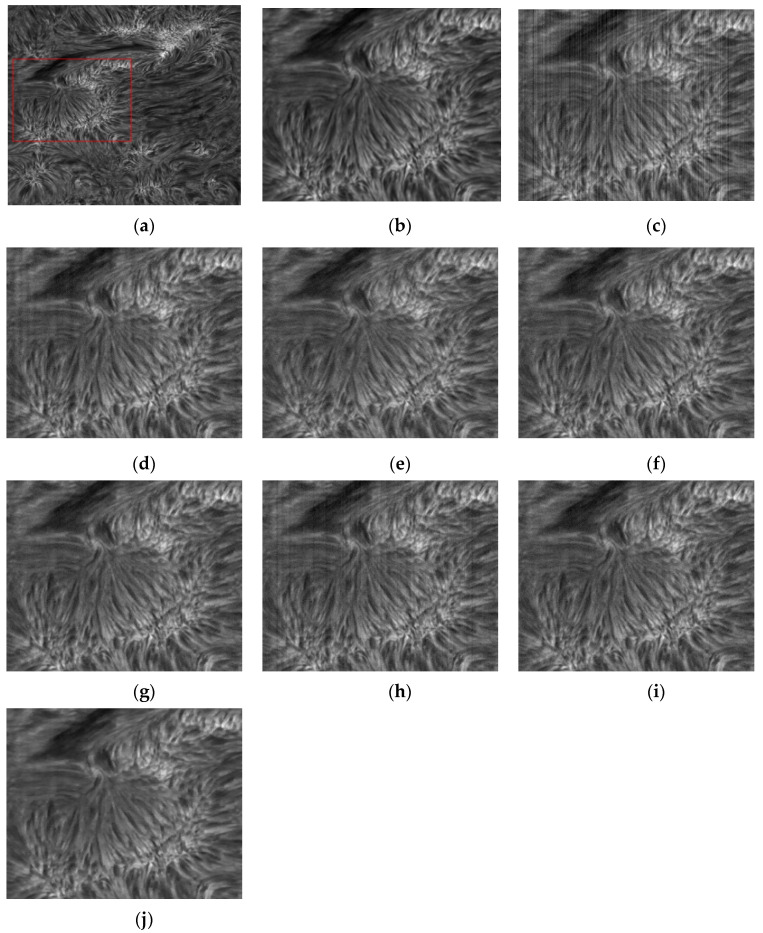
When σ=12, the image of solar active region and the denoising results of various methods. (**a**) Noise image; (**b**) Local area of clear image; (**c**) Local region of noise image; (**d**) wavelet; (**e**) UTV. (**f**) ${ℒ}_{0}$; (**g**) SILR; (**h**) ASSTV; (**i**) GSUTV; (**j**) SUTV.

Judging from the subjective feeling, the Figure 3d,h has obvious residual stripe noise. Figure 3i has a small amount of stripe residue in some areas. Figure 3e,f almost completely remove the stripe noise, but the removal effect of PFPN noise is limited. Careful observation can clearly find that there are obvious random noises in the Figure 3e,f. In the Figure 3g,j the stripe noise is completely removed and the random noise is suppressed to a great extent. The Figure 3g,j has a high similarity with the original clear image.

**Figure 4:**
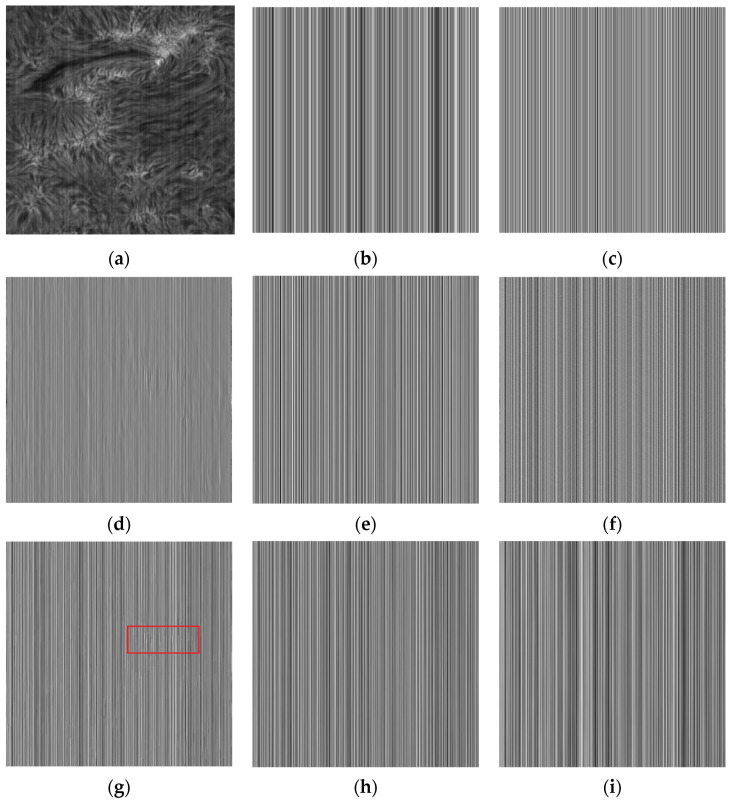
Stripes extracted from active regions by various methods (a) Noise Image; (**b**) Artificially added CFPN; (**c**) wavelet; (**d**) UTV; (**e**)$\;{ℒ}_{0}$; (**f**) SILR;(**g**) ASSTV; (**h**) GSUTV; (**i**) SUTV.

The difference image is the difference between the noise image Y and the filtered result U. The performance of various methods for extracting stripes and whether the difference image contains the structure information of the original image can be clearly observed from the difference image. By observing the difference image in Figure 4, it is found that there are inaccurate estimates of the stripes in Figure 4d,g, and it is obvious that the structure information of the original image is left behind in their difference image. In Figure 4c, although a regular difference image is extracted, the difference image is significantly different from Figure 4b. From the stripe transformation trend, the overall transformation trend of Figure 4e,f,h,i is similar to that of Figure 4b. After careful discrimination, we can also find that Figure 4i is very close to Figure 4b in both trend and intensity of change. In terms of our subjective judgment. Figure 4i has the best fringe extraction effect.

**Figure 5:**
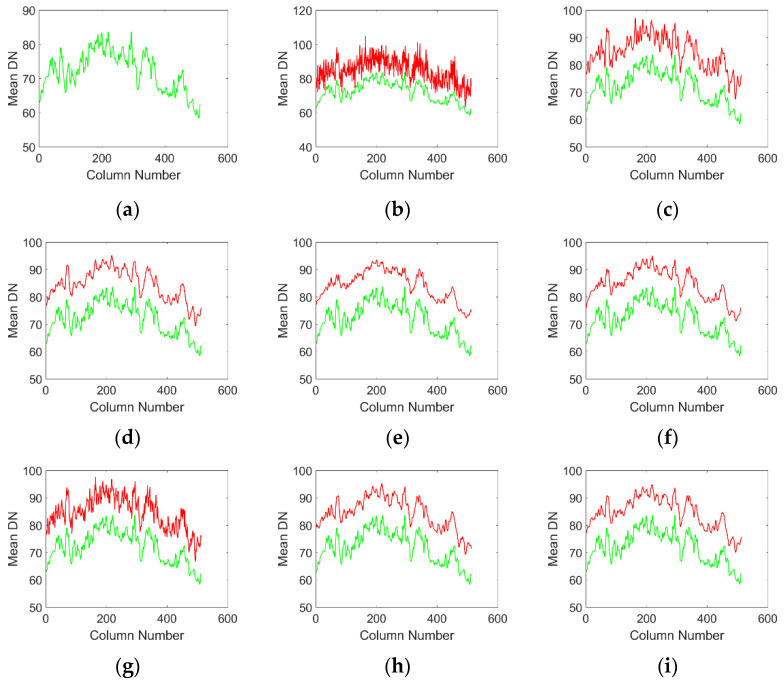
Mean cross-track curves of various denoising results, where the green curve in each result is the mean cross-track curve of the original image, and the red curve is the mean cross-track curve of various denoising results. (**a**) Original picture; (**b**) Noise image; (**c**) wavelet; (**d**)UTV; (**e**) ${ℒ}_{0}$; (**f**)SILR. (**g**)ASSTV; (**h**)GSUTV; (**i**)SUTV.

From the mean cross track curves of denoised images by various methods, mean cross-track curves of Figure 5c,g have obvious noise fluctuations, which reflects that these methods do not completely remove the stripe noise, and still retain the residual stripe in the denoising results. This result is consistent with the subjective visual perception in Figure 3. Figure 5e has obvious over smoothing, which leads to the loss of some details. From the mean cross track curve, Figure 5d,h has a high similarity with the average curve of the clear original image, but there are small fluctuations on the whole curve, which is mainly caused by PFPN. Although these two methods can suppress the stripe noise very well, the suppression effect of PFPN is not good. Figure 5f,i has a high similarity with the original image from the visual point of view, and also suppresses the PFPN noise to a certain extent. It is difficult to evaluate the advantages and disadvantages of the two methods subjectively. Next, we can quantitatively evaluate the advantages and disadvantages of various methods through objective evaluation.

It is difficult to evaluate the advantages and disadvantages of the two methods subjectively. Next, we can quantitatively evaluate the advantages and disadvantages of various methods through objective evaluation

**Figure 6:**
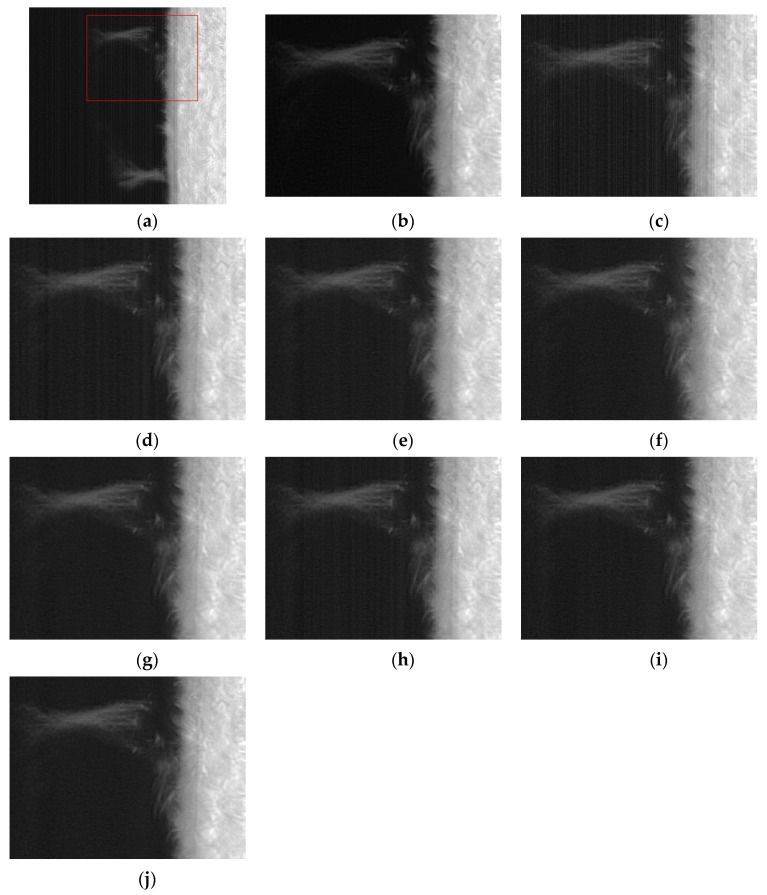
When σ=12, the sun’s photosphere image and the denoising results of various methods. (**a**) Noise image; (**b**) Local area of clear image; (**c**) Local region of noise image; (**d**) wavelet; (**e**) UTV. (**f**) ${ℒ}_{0}$; (**g**) SILR; (**h**) ASSTV; (**i**) GSUTV; (**j**) SUTV.

The sun’s photosphere image shown in Figure 6 is a single image scene with little detail compared to the active area image. Stripes can be easily detected if they are slightly less thoroughly denoised. From the denoising results of various methods in Figure 6, it is shown that there is obvious residual stripe noise in Figure 6d,e,h. From the results of Figure 6f,g,i, although stripe noise is removed from both, significant random PFPN noise residues can be found for both. Overall, Figure 6j has better denoising performance than others, which not only removes stripes but also suppresses random noise to some extent.

Overall, Figure 6j has better denoising performance than others, whereby not only are stripes removed but also random noise is suppressed to some extent.

**Figure 7:**
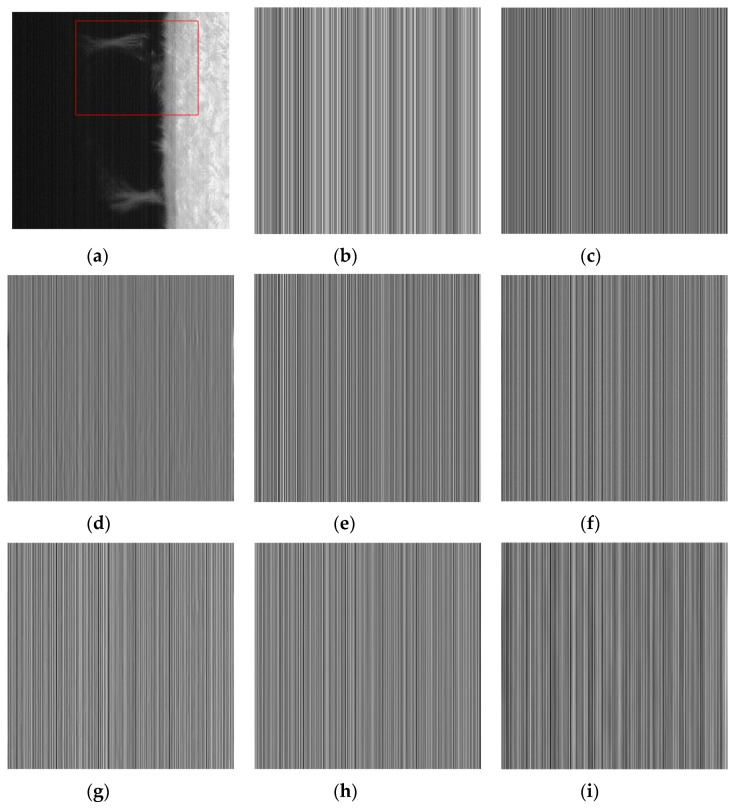
Stripes extracted from photosphere images by various methods. (**a**) Noise Image; (**b**) Artificially added CFPN; (**c**) wavelet; (**d**) UTV; (**e**) L_0; (**f**) SILR; (**g**) ASSTV; (**h**) GSUTV; (**i**) SUTV.

From the difference image itself, the vast majority of the methods extract regular stripe noise patterns. In addition to Figure 7d methods with some residual information of the original image. From the similarity of various results with Figure 7b, the Figure 7f,i has the best similarity with the Figure 7b, both in intensity and pattern.

**Figure 8:**
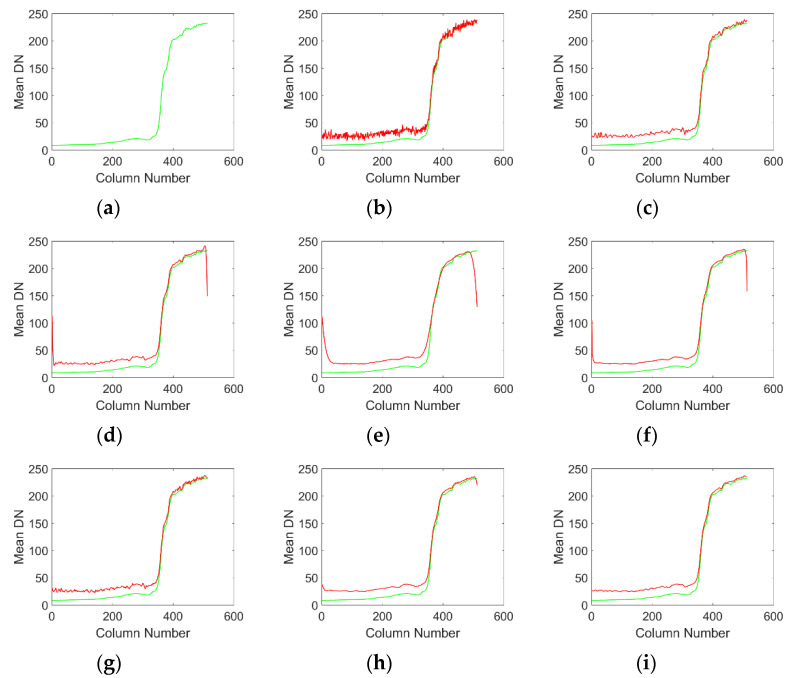
Mean cross-track curves of various denoising results, where the green curve in each result is the mean cross-track curve of the original image, and the red curve is the mean cross-track curve of various denoising results. (**a**) Original picture; (**b**) Noise image; (**c**) wavelet; (**d**) UTV; (**e**) ${ℒ}_{0}$; (**f**) SILR. (**g**) ASSTV; (**h**) GSUTV; (**i**) SUTV.

Similarly, we can subjectively evaluate the noise removal performance of various methods by observing mean cross-track curve of the noise removal results in quiet areas. Figure 8c,g shows the worst image results. As a result of incomplete stripe removal, visible noise residues can be seen on the image. From the result of the curve in Figure 8e, it can be seen that the ${ℒ}_{0}$ method oversuppresses the stripe noise, which leads to the over smoothing of the curve. Figure 8d,f,h,i has the highest visual similarity to the original image and preserves as much detail as possible while stripes are removed. In terms of our subjective vision, it is difficult to accurately distinguish which Figure 8d,f,h,i work best. Maybe different people will get different results. Next, we compare the performance of each method in detail from the objective evaluation index.

#### 3.2.2. Objective Evaluation

**Figure 9:**
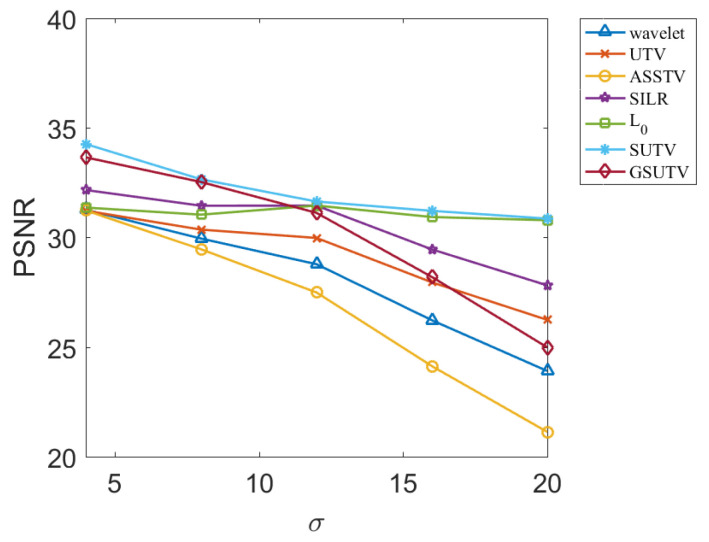
PSNR of denoising results of various methods for solar active region.

**Figure 10:**
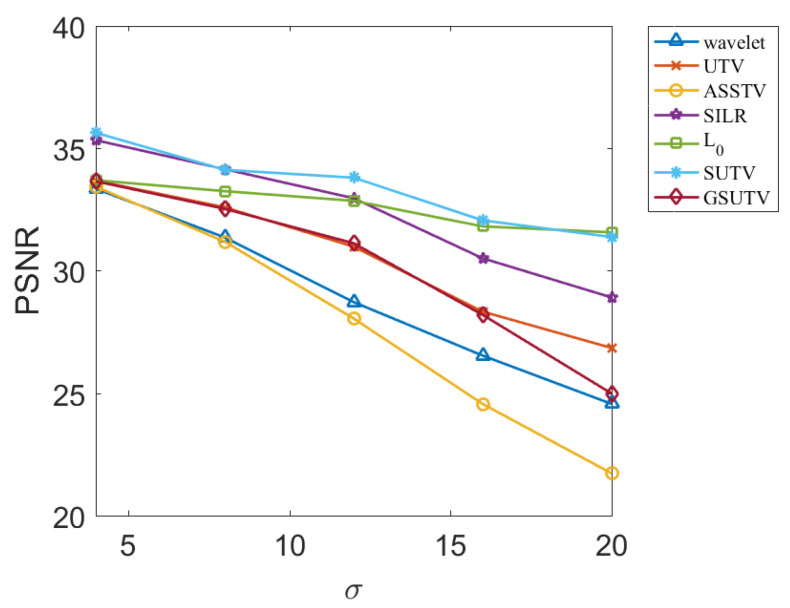
PSNR of denoising results of various methods for solar photosphere.

**Figure 11:**
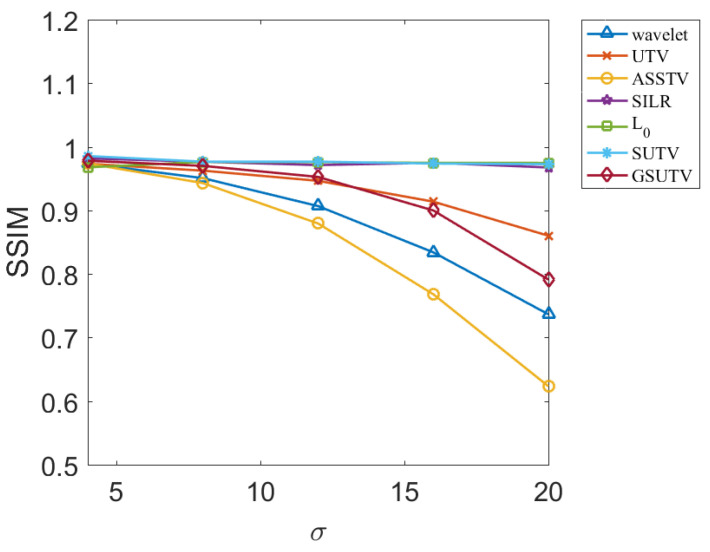
SSIM of denoising results of various methods for solar active region.

**Figure 12:**
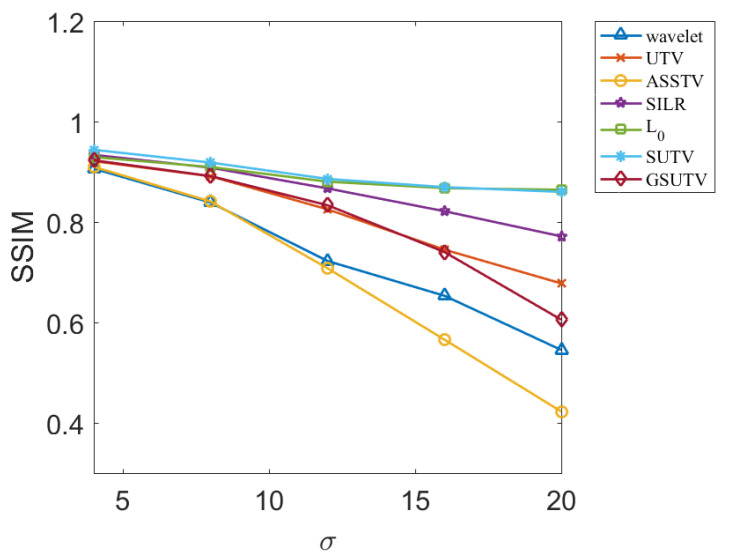
SSIM of denoising results of various methods for solar photosphere.

Next, we objectively evaluate the noise removal performance of each method through two evaluation indexes, PSNR and SSIM, which are relatively objective. PSNR and SSM results for various methods at different image and noise intensities are shown in Figure 9, Figure 10, Figure 11 and Figure 12.

According to the data in Figure 9, Figure 10, Figure 11 and Figure 12, the SUTV method can effectively remove the noise in both the simple photosphere image and the relatively complex active region image. SUTV can get the best PSNR and SSIM value compared with other methods in most of the time. It also fully shows that our method has high robustness and can adapt to different intensity of noise. It should be noted that the regularization parameters used in various methods are adjusted to the optimal value when σ = 12. The regularization coefficients used in denoising other noise intensity images are also determined when σ = 12. The data in Figure 9, Figure 10, Figure 11 and Figure 12 are obtained under such conditions.

### 3.3. Empirical Conclusions

From the experimental results, the PSNR and SSM values of all methods decrease to some extent with the increase of noise intensity. For the SUTV method, PSNR decreased from 35.64 at σ=4 to 31.38 at σ=20 and SSIM from 0.9438 to 0.8598. For solar active area images, PSNR decreased from 34.26 at σ=4 to 30.86 at  σ=20 and SSIM decreased from 0.9855 to 0.9727, but the four curves related to SUTV were at the top of all curves and the rate of decline was even and gentle. This fully demonstrates that SUTV has good adaptability to various noise levels. Most of the other methods show that the denoising effect decreases rapidly with the increase of noise level. This indicates that the regularization parameters of these methods are not well adapted. In order to get better denoising results, we need to adjust parameters repeatedly for different noise levels. From Figure 9, Figure 10, Figure 11 and Figure 12, the method of SUTV not only improves the signal-to-noise ratio of the denoised image, but also guarantees the maximum similarity with the original image. Therefore, SUTV has the most stable noise removal effect.

### 3.4. Real Image

**Figure 13:**
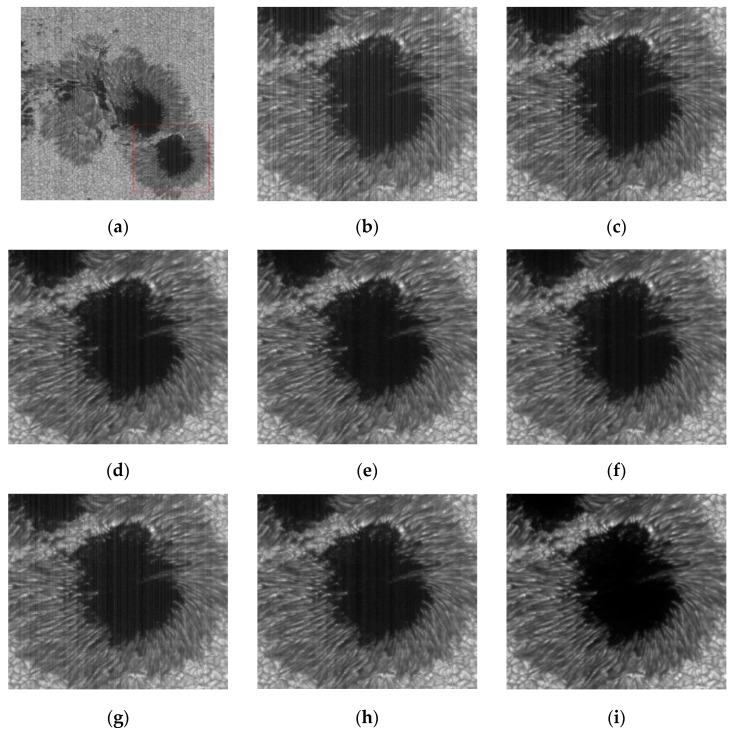
The denoising results of sunspot in solar active region by various methods. (**a**) The image of solar active region is in TiO band; (**b**) Noise Image in Sunspot Area (**c**) wavelet; (**d**) UTV; (**e**) ${ℒ}_{0}$; (**f**) SILR; (**g**) ASSTV; (**h**) GSUTV; (**i**) SUTV.

For the analysis of sunspots in Figure 13c,d,g shows that there is a clear stripe noise almost on the whole image. Figure 13e,f,h removes almost all of the noise except for slightly striped residues in the black area in the middle. The denoising result Figure 13i of the method we recommend is the most thorough one of all. No residual noise stripes can be seen on the whole image.

**Figure 14:**
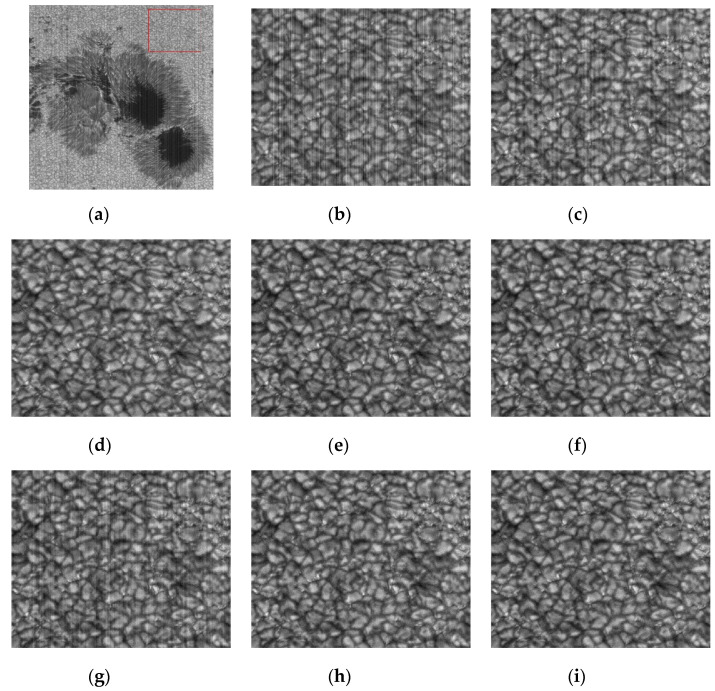
Noise removal results of rice grains in solar active regions by various methods.(**a**) The image of solar active region is in TiO band; (**b**) Noise image in rice grain area (**c**) wavelet; (**d**) UTV; (**e**)$\;{ℒ}_{0}$; (**f**) SILR; (**g**) ASSTV; (**h**) GSUTV; (**i**) SUTV.

From the analysis of the denoising results of the rice granule area in Figure 14, it can be seen that, the stripe noise in Figure 14d–f,h,i is completely removed.

We just need to look carefully to see that there are other problems with their noise removal results. Although the stripes are removed by method d, e, f and h, there is still a significant residual of random noise, which is mainly caused by PFPN. However, the method i removes both the stripe noise and the random noise. This is mainly based on the optimization model. We think that the noise image consists of U, S and N, which not only suppresses the stripe noise S but also suppresses the random noise N. Other methods either consider only the special structural properties of S to suppress or only the random properties. Therefore, they can not remove noise from CMOS images in a comprehensive way. In comparison, method i is the most comprehensive method to consider the noise structure among all the methods involved in the comparison. Therefore, it can not only remove stripe noise, but also suppress random noise.

### 3.5. Discussion

#### 3.5.1. Selection of Parameters

Analysis of the model (5) reveals that the parameters that need to be adjusted are $a_{2},a_{3},a_{4}$ and $w_{2},w_{3},w_{4}$ several parameters, of which $w_{2},w_{3},w_{4}$ are the iteration step needed in the gradient rise method. To simplify the parameter adjustment process we will use the fixed step method, we set $w_{2},w_{3},w_{4}$ to a fixed value of 0.5. However, the adjustment of $a_{2},a_{3},a_{4}$ is the key to achieve good results. The basic criterion for parameter adjustment is to increase the value of $a_{2}$, to increase the weight of horizontal variation and to increase the filter intensity in the horizontal direction if the remaining stripes are obvious in the denoising result. When the difference image between the noise image and the filtered result image has irregular stripes and obvious residual original structure features. At this point we can increase $a_{3}$ Value. When the intensity of random noise caused by PFPN is found to be too high in the denoising result, the $a_{4}$ value can be increased appropriately. According to our debugging experience, the best PSNR can be obtained by adjusting $a_{3}$ at [2.5:4] and $a_{4}$ at [0.2:0.4].We adaptively adjust the $a_{2}$. Because the noise stripes have distinct column characteristics, the appearance of vertical stripes can change the DC component of each column after Fourier transform. This causes a large fluctuation between the DC components of each column. Next, we use the following two steps to complete the adaptive adjustment of $a_{2}$.

First, Fourier transforms are made for each column:

$$F_{:i}=ℱ\left(Y_{:i}\right)$$

where F is the Fourier transform operator, Y is the noise image, and i is the column number.

Secondly, in order to reduce the fluctuation between the DC components of each column, we use the following formula to dynamically adjust $a_{2}$

(16) $$\alpha_{2}=\frac{{\left\|\frac{\partial F_{1:}}{\partial x}\right\|}_{1}}{10^{5}}\cdot C$$

where $\frac{\partial F_{1:}}{\partial x}\;$ represents the horizontal differentiation of the DC component in F. C is a fixed value, which we set to 0.9 based on our debugging experience. When the stripes are more obvious, $a_{2}$ will have a larger value after calculation by Equation (16). However, as the optimization equation iterates, the stripes are gradually suppressed and the ${\left\|\frac{\partial F_{1:}}{\partial x}\right\|}_{1}$ values begin to decrease, thereby reducing the smoothness in the horizontal direction. Other optimization items are dominant at this time. Thus, the dynamic adjustment of parameters can be achieved throughout the process. As a result of the above analysis, the proposed method has better robustness and can achieve good results with different intensity noise.

#### 3.5.2. Running Time

All test procedures are implemented in MATLAB on a desktop personal computer with a 3.4-GHz CPU and 8 GB RAM. By looking at the execution time of each method in Table 1, our proposed SUTV method is not optimal in time. For general CMOS cameras, the change of FPN is a slow process, which is closely related to the ambient temperature and working time.Overall, this change cycle is usually a few hours, which means that FPNs can be approximated as unchanged over a few hours. We can use the extracted FPN as compensation to remove the FPN noise on the image during this time period. Therefore, the tens of seconds spent on a single FPN extraction are negligible relative to hours. 

## 4. Summary

Most stripe removal methods based on optimal stripe removal only remove stripes from the image itself, but little consideration is given to the special directional structure of the stripes. Although some scholars have considered the structural characteristics of the stripes, they have considered less the random noise generated by PFPN. Therefore, in their denoising results, although the stripe noise is effectively removed, significant random noise remains. Our method is a more comprehensive method of noise removal, taking into account all forms of noise. At the same time, the adaptive regular parameter adjustment strategy reduces the difficulty of parameter adjustment to a certain extent. The application of this method can adapt to different intensity noise images and improve the robustness of noise reduction to a certain extent. Then there is a drawback to our method, which has a more complex model than other optimization methods and requires parameter adjustments to be computed, resulting in longer code runtime. In the end, we will further optimize the model and further speed up the calculation efficiency.

## Figures and Tables

**Table 1:** Running time of various methods. Units are seconds. The iteration period is 150 times.

Size	WAFT	UTV	ASSTV	SILR	**${ℒ}_{0}$**	GSUTV	SUTV
512 × 512	0.1196	4.9194	18.0126	27.9707	20.2170	24.9707	29.4003
